# Omega‐6/Omega‐3 Ratio in Dementia Prevention ‐ Study Design and preliminary data of a feasibility study on lifestyle intervention

**DOI:** 10.1002/alz.090716

**Published:** 2025-01-09

**Authors:** Ayda Rostamzadeh, Lena Sannemann, Lara Bohr, Michelle Gerards, Sarah Egert, Alfredo Ramirez, Frank Jessen

**Affiliations:** ^1^ Department of Psychiatry and Psychotherapy, Medical Faculty, University of Cologne, Cologne Germany; ^2^ Institute of Nutritional and Food Sciences, Nutritional Physiology, University of Bonn, Bonn, NRW Germany; ^3^ Cluster of Excellence Cellular Stress Responses in Aging‐Associated Diseases (CECAD), University of Cologne, 50931, Cologne Germany; ^4^ Deutsches Zentrum für Neurodegenerative Erkrankungen e. V. (DZNE) Bonn, Bonn Germany; ^5^ Division of Neurogenetics and Molecular Psychiatry, Department of Psychiatry and Psychotherapy, University of Cologne, Medical Faculty, Cologne Germany; ^6^ Excellence Cluster on Cellular Stress Responses in Aging‐Associated Diseases (CECAD), University of Cologne, Cologne Germany

## Abstract

**Background:**

Alzheimer’s disease (AD) is the most common etiology of dementia. As the progression of the disease may be slowed down by disease‐modifying therapies, but not stopped, research identifying further therapeutic approaches is necessary. Due to the multifactorial etiology of AD, targeting modifiable risk factors for dementia, including diet, is a starting point for preventive interventions. Currently, dementia prevalence is highest in North America and Western Europe, which correlates with an excess of saturated fat in the Western diet (WD). How precisely fatty acid dietary composition contributes to AD pathogenesis is poorly understood. Polyunsaturated fatty acids (PUFA), which account for 18% of total fat intake, include the essential fatty acids ω‐6‐linolenic and ω‐3‐alpha‐linolenic acid, which cannot be synthesized de novo and must be consumed in the diet. Omega ω‐6 and ω‐3 PUFA are found in high concentrations in the brain. Current findings indicate that a high ω‐6/ω‐3 ratio may contribute to a dysbalance between pro‐ and anti‐inflammatory processes and consequently accelerate or trigger neurodegenerative processes.

**Method:**

This randomized‐controlled feasibility study investigates the effects of a 10‐week dietary intervention in a sample (*n* = 60) of cognitively healthy individuals from the Cologne Dementia Prevention Registry, who follow a WD. The dietary intervention consists of both a change in diet to reduce ω‐6 PUFA and supplementation with fish oil capsules to increase ω‐3 PUFA intake. The primary endpoint of the study is the reduction of the ω‐6/ω‐3 ratio, measured in serum phospholipids, including levels of ω‐6 and ω‐3 fatty acids. Secondary, exploratory endpoints include changes in dietary behavior, cognitive performance, markers of inflammation and oxidative status, epigenetic analyses (DNA methylation), and changes in lifestyle and well‐being.

**Result:**

We present the rationale, design and preliminary results of a 10‐week dietary intervention trial in a cognitively healthy cohort.

**Conclusion:**

This study investigates the feasibility and effects of a lifestyle‐based intervention on the ω‐6/ω‐3 ratio and further biological and cognitive‐behavioral secondary outcomes. The results can inform future trials aimed at a deeper mechanistic understanding of the reduction of the ω‐6/ ω‐3 ratio in humans; eventually targeting clinical primary outcomes, such as cognition, over time.

